# Establishing the effectiveness of patient decision aids: key constructs and measurement instruments

**DOI:** 10.1186/1472-6947-13-S2-S12

**Published:** 2013-11-29

**Authors:** Karen R Sepucha, Cornelia M Borkhoff, Joanne Lally, Carrie A Levin, Daniel D Matlock, Chirk Jenn Ng, Mary E Ropka, Dawn Stacey, Natalie Joseph-Williams, Celia E Wills, Richard Thomson

**Affiliations:** 1 Harvard Medical School and General Medicine Division, Massachusetts General Hospital, 50 Staniford Street, 9th floor, Boston, Massachusetts, 02114, USA; 2 Women’s College Research Institute, Women's College Hospital, 790 Bay Street, Room 728, Toronto, Ontario, M5G 1N8, Canada; 3 Institute of Health and Society, Newcastle University, Baddiley-Clark Building, Richardson Road, Newcastle upon Tyne, NE2 4AX, UK; 4 Informed Medical Decisions Foundation, 40 Court Street, Boston, Massachusetts, 02108, USA; 5 School of Medicine, University of Colorado, 12631 E 17th Avenue, Aurora, Colorado, 80045, USA; 6 Department of Primary Care Medicine, University of Malaya, 50603, Kuala Lumpur, Malaysia; 7 School of Medicine, University of Virginia, P.O. Box 800717, Charlottesville, Virginia, 22908-0717, USA; 8 Ottawa Hospital Research Institute, University of Ottawa, 501 Smyth Road. Ottawa, Ontario, K1H 8L6, Canada; 9 Institute of Primary Care and Public Health, Cardiff University, 2nd Floor, Neuadd Meirionnydd, HeathPark, Cardiff, CF14 4YS, UK; 10 Ohio State University, 384 Newton Hall, 1585 Neil Avenue, Columbus, Ohio, 43210, USA

## Abstract

**Background:**

Establishing the effectiveness of patient decision aids (PtDA) requires evidence that PtDAs improve the quality of the *decision-making process* and the quality of the choice made, or *decision quality*. The aim of this paper is to review the theoretical and empirical evidence for PtDA effectiveness and discuss emerging practical and research issues in the measurement of effectiveness.

**Methods:**

This updated overview incorporates: a) an examination of the instruments used to measure five key decision-making process constructs (i.e., recognize decision, feel informed about options and outcomes, feel clear about goals and preferences, discuss goals and preferences with health care provider, and be involved in decisions) and decision quality constructs (i.e., knowledge, realistic expectations, values-choice agreement) within the 86 trials in the Cochrane review; and b) a summary of the 2011 Cochrane Collaboration’s review of PtDAs for these key constructs. Data on the constructs and instruments used were extracted independently by two authors from the 86 trials and any disagreements were resolved by discussion, with adjudication by a third party where required.

**Results:**

The 86 studies provide considerable evidence that PtDAs improve the decision-making process and decision quality. A majority of the studies (76/86; 88%) measured at least one of the key decision-making process or decision quality constructs. Seventeen different measurement instruments were used to measure decision-making process constructs, but no single instrument covered all five constructs. The Decisional Conflict Scale was most commonly used (n = 47), followed by the Control Preference Scale (n = 9). Many studies reported one or more constructs of decision quality, including knowledge (n = 59), realistic expectation of risks and benefits (n = 21), and values-choice agreement (n = 13). There was considerable variability in how values-choice agreement was defined and determined. No study reported on all key decision-making process and decision quality constructs.

**Conclusions:**

Evidence of PtDA effectiveness in improving the quality of the decision-making process and decision quality is strong and growing. There is not, however, consensus or standardization of measurement for either the decision-making process or decision quality. Additional work is needed to develop and evaluate measurement instruments and further explore theoretical issues to advance future research on PtDA effectiveness.

## Background

As outlined in the introductory paper in this series of manuscripts, 12 core dimensions for the assessment of PtDAs were generated in 2005 by the International Patient Decision Aids Standards (IPDAS) Collaboration, and further reinforced by an extensive Delphi survey undertaken by the IPDAS Collaboration [1]. One of these 12 quality dimensions is the measurement of the effectiveness of a patient decision aid (PtDA).

To establish the effectiveness of a PtDA, it is critical to provide evidence that the PtDA improves two constructs: i) the *quality of the decision-making process* and ii) the *quality of the choice that is made* (i.e., “decision quality”). In the IPDAS Collaboration’s original 2005 Background Document [2], the chapter on establishing the effectiveness set out key attributes for both the decision-making process and decision quality; and that work subsequently prompted the Cochrane Collaboration’s Systematic Review of Decision Aids to redesign the presentation of their results to follow these constructs and attributes.

For the *quality of the decision-making process*, the core attributes that should be measured include the extent to which PtDAs help patients to:

• Recognize that a decision needs to be made (e.g., as measured by items in the Preparation for Decision Making Scale (PMDS) [3].

• Feel informed about the options and about the risks, benefits, and consequences of the options (e.g., as measured by the “Feeling Uninformed” subscale of the Decisional Conflict Scale[4]).

• Be clear about what matters most to them for this decision (e.g., as measured by the “Unclear Values” subscale of the Decisional Conflict Scale (DCS) [4]).

• Discuss goals, concerns, and preferences with their health care providers (e.g., as measured by items in the Perceived Involvement in Care Scale (PICS) [5]).

• Be involved in decision making (e.g., as measured by the Control Preferences Scale (CPS) [6] and adaptations of the CPS).

The quality of the choice that is made, or *decision quality*, is defined as the extent to which patients are informed and receive treatments that reflect their goals and treatment preferences [1,7]. It follows from this construct definition that two core attributes should be measured:

• Informed patient: This attribute is measured by assessing a patient’s knowledge of the options and outcomes. It is not assessed in terms of patient perceptions of their knowledge level; instead, factual items are used to assess objectively a patient’s understanding of the information. This may, when applicable, include an assessment of whether or not the patient holds realistic expectations of risks and benefits.

• Concordance between what matters most to the patient and the chosen option: Most approaches to measuring this attribute require (1) the elicitation of a patient’s goals and/or treatment preferences; (2) the identification of the patient’s chosen or implemented option; and (3) a calculation of the extent to which the option best meets the patient’s stated goals or treatment preferences.

These two constructs—the quality of the decision-making process and the quality of the decision—are equally relevant to PtDAs that address treatment as well as screening decisions in which there are two or more reasonable options. They are also applicable to other settings—for example, in chronic disease when patients are facing choices (e.g., whether or not to start a statin for patients with diabetes).

Decisions to enact lifestyle changes that have a significant behavior change component (such as smoking cessation or weight loss) may require different or additional support (e.g., supported self-management or motivational interviewing). Since the approaches used in these kinds of health care situations often are not PtDAs, the measures of effectiveness for these situations are not covered in this review.

We note that other constructs—as either process or outcome variables—have been used to evaluate the effectiveness of PtDAs, such as decision self-efficacy, decision regret, patient satisfaction with decision making, and treatment choice. Furthermore, there are many survey instruments and scales that cover one or more attributes within the decision-making process and decision quality constructs. However, in this paper we focus on the five decision-making process and the two decision quality attributes that are summarised above. Our aims are to discuss the theoretical justification for using these constructs—and their attributes—when evaluating the effectiveness of PtDAs, to review current empirical evidence on measurement of PtDA effectiveness (considering the different measurement instruments that have been used to assess these key constructs), and to highlight notable practical and research issues in measurement that emerged from this analysis.

## Theoretical justification for evaluating patient decision aids on this quality dimension

### Scientific rationale

Establishing the effectiveness of any health care intervention, including PtDAs, is critical. There is considerable consensus that PtDAs should: a) improve the quality of the decision-making process; and b) increase decision quality or the likelihood that individuals choose and/or receive health care interventions that are most consistent with their informed and considered values[1,8-13]. The Cochrane Collaboration’s Systematic Review of Decision Aids reports their results according to these key constructs and has found considerable evidence that PtDAs improve these outcomes [14]. However, the field needs to continue to generate high-quality evidence regarding the benefits and harms of PtDAs, as well as their impact in vulnerable populations, and in a range of health conditions and healthcare systems, including different countries and cultures. We have focused on the results of randomized controlled trials, drawing extensively on those incorporated within the Cochrane Collaboration’s review [14], as the gold standard for assessment of interventions.

### Ethical rationale

PtDAs are viewed as a means of shifting from paternalism to increased patient engagement in decision making, including shared decision making (SDM), in healthcare. SDM is a process by which a decision is made between a patient (and their families or others), and one or more healthcare professionals. It offers a model to improve patient engagement, particularly in preference-sensitive decisions in which there are multiple reasonable options and in which the choice should be influenced by patient goals and preferences.

As well as an ethical imperative to engage patients in decisions about their own care, PtDAs can support improvements in informed consent. King and colleagues (2006) have argued that traditional informed consent methods are inadequate to engage and inform patients about treatment options in preference-sensitive decisions [15]. SDM goes beyond information-giving to supporting the formulation and communication of informed preferences. Thus, SDM may offer an ethically and legally supported means for fostering informed choice, including transparent presentation of potential benefits and harms. When usual care is compared to the use of PtDAs, usual care has been shown to be inadequate for ensuring that patients are informed and have realistic expectations [14].

The PtDA literature is relatively light on the exploration of adverse effects, although such effects have been posited. Adverse effects might include, for example, an increase in inequalities (through being more accessible to, or used by, well-educated patients), or increased conflict with public health priorities (through selection of “less effective” interventions) [16]. For example, well informed patients may select to forego colon cancer screening or other interventions that have been shown to be effective in prolonging life or other outcomes. To the extent that “pay-for-performance” or other quality measurement initiatives for health care providers or health systems focus on the public health priorities as opposed to the individual’s informed choices, this may result in increased tension and conflict with the goals of PtDAs. Other adverse effects might include increased patient anxiety when patients are faced with clinical uncertainty[17], or are offered an unexpected role in decision making and are initially wary of engaging in decision making [18], or feel unsupported or ‘abandoned’ if decision making is not actually shared but is unduly delegated to patients[19]. Adverse effects could also occur if PtDAs are not well-developed, or become out-of-date, and therefore might bias decisions.

### Conceptual rationale

Measures of the “*quality of the decision-making process*” and “*decision quality*” highlighted in this manuscript have underpinnings in theories of decision making. Normative theories of decision making, such as subjective expected utility theory, are based on the ideal that patients approach decisions rationally and are able to weigh the risks and benefits of all available interventions [20]. Descriptive theories of decision making, such as prospect theory, demonstrate that humans are subject to cognitive biases that cause decision making to deviate from the normative/rational ideal [21]. For example, a well-known cognitive bias has to do with the effects of framing, where people tend to be risk-averse when statistics are presented as gains and risk-seeking when they are presented as losses [22]. These kinds of biases can threaten a person’s ability to acquire accurate knowledge or to make a decision concordant with their values, thus threatening the quality of their decision. The Dual-Process Theory of decision making argues that people make decisions either “intuitively” (i.e., quickly drawing on past experiences), or “reasonably” (i.e., using a thoughtful, analytic approach), with the latter being less subject to many of the cognitive biases[21]. PtDAs are designed to encourage a more deliberative decision-making process that can help to minimize cognitive bias. If a more reasoned, normative approach is pursued, it follows that the actual choice is more likely to be informed and value concordant, resulting in higher “decision quality.”

Although conceptually many of these theories share similar underpinnings (e.g., an emphasis on information and the use of deliberative processes to align choices with goals), there is considerable debate about how that is operationalized into specific measures. The debate involves not only how to measure the construct (e.g., measuring patients’ preferences using the standard gamble in formal decision analysis versus using attitude scales), but also when these are measured. For example, Elwyn and Miron-Shatz (2010) have argued that it is best to measure the quality of the decision-making process before and immediately after exposure to the PtDA. They argue against retrospective assessments that are subject to hindsight bias, particularly following adverse clinical outcomes, and might distort the assessment of the decision-making process [23]. Others point to theories, such as differentiation and consolidation theory, that suggest that patients will continue to react to and interpret the decision after it is taken and that it is important to measure regret and other variables after the decision has been made [24].

Which outcomes to measure are also being debated. A key goal of health care is to improve health outcomes, and many outside the field ask about the impact of PtDAs on health outcomes. There are several challenges to the use of clinical health outcomes (such as pain, overall quality of life, or mortality) to assess the effectiveness of PtDAs. First, the nature of the situation addressed by PtDAs requires that there are multiple reasonable options, often with different potential effects (positive or negative) on health outcomes. Thus, by definition, there is usually not one clearly superior treatment or intervention. Second, many of these decisions are made under uncertainty, and are essentially making a bet. The appropriate evaluation of a bet depends on the odds, not the outcome. For example, a patient may choose to have surgery, feeling that the benefits outweigh the harms, and yet may suffer a severe, unanticipated complication during the procedure. This bad outcome should be used to improve the delivery of the procedure, but it should not reflect poorly on the decision to have surgery. A third challenge with using health outcomes as a measure is that this often requires setting a global standard (e.g., longer life is always better, or less pain is always better). However, studies have shown that patients vary in their willingness to trade off quality of life and quantity of life. For example, some patients may elect to forego chemotherapy if their desire to avoid short-term severe side effects outweighs their desire for increasing short-term survival. In sum, to the extent that patients feel differently about potential health outcomes, it is necessary to measure the effectiveness of PtDAs by the extent to which they enable patients to achieve the outcomes they most desire while also avoiding those they most dislike.

### Policy rationale

There are several recent, widespread health policy drivers across several different countries that focus on patient engagement, PtDAs, and SDM, emphasizing the need for a robust evidence base. For example, in the United States, the Institute of Medicine and the National Priorities Partnership (NPP) have identified patient and family engagement and patient-centered care—defined in part as ensuring that patient are informed, meaningfully involved in treatment decisions, and receive treatments that reflect patients’ goals, needs, and preferences—as one of six national health care priorities [25,26]. This has had wide impact; for example, prompting the National Quality Forum (NQF), the US body that endorses performance measures, to examine their endorsed measures: none were related to SDM, and hence the NQF has identified SDM as a priority area for additional measure development [27].

Similarly, SDM (“Nothing about me, without me”) is included within the latest UK Government health policy [28], and is embedded in legislation [29]. The Department of Health has commissioned an extensive program of development of PtDAs [30]. Further description on policy developments internationally can be found in a special 2011 issue of The German Journal for Quality in Healthcare [31].

The policy perspective creates pressure to measure an additional variable — costs. If health systems are to fund access to PtDAs, then they want to know the intervention is not only effective but also cost-effective. The impact of PtDAs on utilization has been demonstrated in a small number of decisions, with patients less likely to select more invasive options where such choices exist (e.g., active surveillance or surgery for benign prostate disease) [14]. A recent large demonstration project in the US found that widespread use of decision aids in hip and knee osteoarthritis led to reduced surgical rates and reduced costs [32]. However, the impact on cost seems to depend on baseline utilisation [28]. Hence, cost alone is neither a sufficient nor an appropriate core measure for effectiveness, which should be based upon improved quality of the decision-making process and decision quality. Nevertheless, as implementation efforts expand, examining the impact on costs and developing more sophisticated assessment of cost-effectiveness, based on appropriate outcomes, will be increasingly important.

## Empirical evidence

### Methods

Data were used from the 2011 Cochrane Collaboration systematic review of PtDAs to assess what is known about the impact of PtDAs on the *quality of the decision making process* and the *quality of the choice that is made* (i.e., *decision quality*) [14]. This review included 86 randomized controlled trials (RCTs) comparing individual PtDAs for treatment or screening decisions to usual care and/or alternative interventions.

We extended the Cochrane Collaboration’s review by gathering additional information about the measurement instruments used to assess key outcomes in each of the 86 studies. Two reviewers independently abstracted information, such as the medical condition covered, mode of administration of the measurement instruments, and whether or not the instrument covered any of the five decision-making process attributes (i.e., recognize decision, feel informed, clear values, discuss goals with health care provider, be involved) or the two decision quality attributes (i.e., knowledge (including realistic expectations), and concordance). Detailed narrative data on the instruments and how they were used were also collected. We compared the abstracted data from each reviewer and reconciled any discrepancies by consulting the full text of the article. The lead authors (KS and RT) adjudicated differences across reviewers that were not able to be resolved by consulting the full text.

In many cases, the actual items from the measurement instruments were not included in the articles, which made it difficult to code accurately. If an instrument was used in more than one study, additional articles were retrieved and reviewed in order to determine whether or not items covered one or more of the attributes. If any of the items included in the instrument elicited information on a particular attribute, we considered it covering that attribute, even if it was not reported on separately. For example, if a study only reported the total score for the Decisional Conflict Scale, we still considered that it covered two of the decision-making process attributes: “feel informed about options, risks and benefits” and “be clear about values.”

The data were collected in structured Excel spreadsheets and after the data were reconciled they were entered into an SPSS file (IBM SPSS Statistics, version 20.0) for analysis. Descriptive statistics were used to examine the frequency of use of the different attributes and measurement instruments.

### Frequency of inclusion of key outcomes in decision aid studies

We abstracted 180 cases in which a reported outcome mapped onto one or more of the decision-making process or decision quality attributes. The majority of studies in the Cochrane Collaboration’s review (76/86; 88%) reported on one or more outcomes that assessed decision-making process or decision quality attributes. On average, each study reported on 2.1 measurement instruments that captured one or more of the attributes (ranging from 0 to 5 instruments per study). Most of the studies that did not report on any of these core attributes only reported the impact on choices or uptake of treatment, without any examination of whether the change in rates reflected an increase or decrease in concordance.

### Measures of the quality of the decision-making process

Our review identified 17 different measurement instruments used to assess aspects of the decision-making process. The most common was the Decisional Conflict Scale (DCS) [4], used in 47 studies, followed by adaptations of the Control Preferences Scale (CPS) [33], used in nine studies. All other instruments were used in four studies or fewer. The other named instruments that covered one or more of the decision-making process attributes included the Autonomy Preference Index (API) [34], COMRADE [35], Decision Satisfaction Inventory (DSI) [36], Perceived Decision Control [37], Perceived Involvement in Care Scale (PICS) [5], Preparation for Decision Making Scale (PDMS) [3], Problem Solving Decision Making Scale (PSDM) [38], Satisfaction with Decision Making Process (SDMP) [39], and Satisfaction with Decision (SWD) [40]. Although the vast majority of trials used patient surveys, one used qualitative methods, involving the coding of audio-taped consults, to assess the level of Informed Decision Making [41].

We did not find a single measurement instrument that covered all of the decision-making process attributes. The Preparation for Decision Making Scale covers the most, and includes items that cover four of the five attributes (it does not have items that assess the extent to which patients feel informed about options and outcomes) [42]. Table 1 shows how often each decision-making process attribute was measured across the 86 studies, an example of an item used to measure the attribute, and the named measurement instruments used. The attributes of “feeling informed about options and outcomes” and “clear about values” are the most commonly assessed (mainly assessed using the DCS), and whether patients “recognize that there is a decision to be made” and whether they “discuss their goals and preferences with their healthcare provider” are the least commonly assessed.

**Table 1:** Frequency of Measurement of Decision-making Process Constructs and Sample Items

	CONSTRUCTS				
	**Recognize Decision**	**Feel Informed**	**Feel Clear about Values**	**Discuss Goals with HCP**	**Be Involved**

**Frequency of use**	13	55	56	8	33

**Unique measurement instruments**	6	5	5	5	20

**Survey instrument, sample item, and response set**	PDMS: Did this educational material help you realize that a decision needs to be made? (“Not at all” to “A great deal”)	DCS: I understand the options for treatment of X. (“Strongly agree” to “Strongly disagree”)	DCS: It is clear which benefits and harms matter most to me. (“Strongly agree” to “Strongly disagree”)	PICS: My doctor encouraged me to talk about my personal concerns related to my medical symptoms (“Strongly agree” to “Strongly disagree”)	CPS: Please select the option that reflects how you would like your medical decisions to be made: • I prefer to make the final decision • I prefer to make the final decision after seriously considering my doctor’s opinion • I prefer that my doctor and I share responsibility for the decision • I prefer that the doctor makes the decision after he/she seriously considers my opinion • I prefer my doctor to make the decision

**Named measurement instruments used**	SWD (n=4) PICS (n=3) PDMS (n=2)	DCS (n=45) SWD (n=4) SDMP (n=4) DSI (n=1)	DCS (n=46); SWD (n=3); SDMP (n=3); PDMS (n=2)	PICS (n=3); PDMS (n=2)	CPS (n=9); SDMP (n=3); API (n=2); COMRADE (n=1); DSI (n=1); PSDM (n=1); PDC (n=1)

*Legend:* HCP=health care provider; PDMS=Preparation for Decision Making Scale; DCS=Decisional Conflict Scale; PICS=Perceived Involvement in Care Scale; CPS=Control Preferences Scale (including adaptations); SWD=Satisfaction with Decision; SDMP=Satisfaction with Decision Making Process; API=Autonomy Preference Index; DSI=Decision Satisfaction Inventory; PSDM=Problem Solving Decision Making Scale; PDC=Perceived Decision Control.

### Impact of PtDAs on decision-making process

The Cochrane Collaboration’s review presents pooled data on three of this constuct’s key attributes, and reports that PtDAs result in: a) a reduction in feeling uninformed (n = 17; mean difference = -6.4 of 100; 95% CI -9.2 to -3.7) assessed with the “Feeling Uninformed” subscale of the Decisional Conflict Scale; b) a reduction in feeling unclear about personal values (n = 14; mean difference = -4.8; 95% CI -7.2 to -2.4) assessed with a subscale of the DCS; and c) reduction in provider controlled decision making (n = 11; RR = 0.61; 95% CI 0.5 to 0.8). There were no data reported in the Cochrane Collaboration’s review on the effectiveness of PtDAs in helping patients to recognize that a decision needs to be made, or to discuss values and preferences with their health care provider [14].

### Measures of decision quality

Across the 86 studies, we found 59 cases in which measurement instruments assessed patients’ knowledge and 21 that included items that assessed realistic expectations. Most of the knowledge questionnaires were multiple choice or true-false in format, and only two were “named” measurement instruments (Breast Cancer Information Test [43] and Breast Cancer Prevention Questionnaire [44]). More than half (36/59, or 60%) of the knowledge instruments were created anew for the study.

Fewer studies (n = 13) reported on concordance or values-choice agreement. Six of these used the Multidimensional Measure of Informed Choice (MMIC) [45-50]. This approach differed from study to study, but generally included an assessment of knowledge (those who score above a level set *a priori* were considered informed), clear values (e.g., those who score 25 or less on the “Unclear Values” subscale of the DCS), and clear intention (those patients who were able to state a clear treatment preference as opposed to being unsure). A composite score of informed choice indicated the percentage of patients who were informed, had clear values, and a clear intention.

The other seven studies used different approaches to calculate concordance. Two of the studies used a straightforward approach and measured concordance by calculating the percentage of patients who received treatment that matched the patients’ stated treatment preference [51,52]. Frosch (2008) used one item to represent men’s preferences, namely their concern about the risk of dying of prostate cancer, and then looked at whether the mean scores on that item differed between men who did or did not get screened for prostate cancer (and then by intervention and control groups) [53]. O’Connor (1999) and Legare (2008) used more sophisticated modeling analyses to calculate concordance [54,55]. Both studies elicited patients’ personal goals and then used a regression model to examine the extent to which the values (independent variables) explained the choices (dependent variable). Although all seven of these studies also captured patients’ knowledge, none of them created a composite decision quality score that reported both informed and concordant choices.

### Impact of PtDAs on decision quality

The Cochrane Collaboration’s review results indicate that: PtDAs improve knowledge by about 14% (a mean difference of 13.8 out of 100; 95% CI 11.4 to 16.2; n = 26 studies), with greater knowledge gains with more complex PtDAs; and improve realistic expectations by 74% (relative risk 1.7; 95% CI 1.5 to 2.1; n = 14 studies), more so when the probabilities are expressed in numbers than words. PtDAs also result in fewer people being undecided, in that more have clear treatment preference (RR 0.6; 95% CI 0.4 to 0.7; n = 10 studies); and, in the presence of explicit values clarification, improve the percentage of informed, values-based choices by 25% (RR 1.3; 95% CI 1.1 to 1.5; n = 8 studies) [14].

## Discussion

The original 2005 publication of the IPDAS Collaboration’s standards highlighted the importance of measuring the decision-making process and decision quality in order to understand the effectiveness of PtDAs. The leaders of the Cochrane Collaboration’s systematic review of decision aids then used those constructs as an organizing framework for reporting their subsequent results. Since 2005, many new randomized controlled trials that test the effectiveness of PtDAs have been published, and this growth of the evidence has strengthened the initial conclusions and added some new findings.

Many of the findings incorporated in the relevant chapter on effectiveness that appears in the IPDAS Collaboration’s 2005 Original Background Document are reconfirmed. With respect to the quality of the decision-making process, PtDAs reduce decisional conflict with regard to feeling uninformed and unclear about personal values, and result in more patients playing an active role in decision making. With respect to core domains of decision quality, PtDAs improve people's knowledge regarding options (patients are more informed) and, when outcome probabilities are included, PtDAs result in more realistic expectations of risk and benefit.

The recent Cochrane Collaboration’s review includes additional evidence for decision quality, with new studies showing that, when compared to simple decision aids, those with explicit values clarification increase the percentage of patients who make an informed, values-based choice. However, the bulk of the studies (6/8) in this meta-analysis used the Multidimensional Measure of Informed Choice approach. Some of these studies used a variation of MMIC that combines knowledge scores, scores on the values subscale of the Decisional Conflict Scale, and the percentage of patients able to state clear treatment preference. It is possible that these findings are simply reaffirming, in a composite measure, that PtDAs improve knowledge and reduce decisional conflict. Although having a clear intention is important, some of the studies using MMIC do not provide direct evidence of increased concordance between patients’ goals and treatment choices.

There is still limited evidence on two key attributes of the decision-making process construct—whether decision aids help patients recognize a decision needs to be made, and whether PtDAs help patients discuss their goals and concerns with their health care providers. Although some survey instruments do contain one or more items that cover these attributes, none reported on them separately and, as a result, these could not be included in the Cochrane Collaboration’s review.

More recent advances in decision quality measurement are not yet reflected in the Cochrane Collaboration’s review. For example, Sepucha and colleagues have published psychometric analyses of three decision quality instruments (for osteoarthritis of the knee or hip, herniated disc, and breast cancer surgery) that assess the extent to which patients are informed and receive treatments that match their goals [56-58]. In general, these instruments meet several criteria for patient-reported outcomes, including test-retest reliability, content and discriminant validity, acceptability, and feasibility. It will be important to include these measurement instruments in trials of PtDAs in order to advance our ability to measure decision quality.

There are strengths and limitations of this review worth noting. Our review has drawn upon robust studies of effectiveness of PtDAs to examine the measurement of major attributes of two core constructs—the quality of the decision-making process and decision quality. Two expert reviewers independently extracted data, with reference to a third reviewer for any disagreements, and with reference to source articles for instruments where necessary. The restriction to measures used in RCTs, however, has resulted in some limitations. First, the review of measurement instruments may have missed instruments that were not used in RCTs in the Cochrane Collaboration review (see, for example, Scholl et al. for a review of measures of SDM)[59]. Second, the RCTs are not designed to provide details about the active ingredients of the interventions and about the contextual factors that might act as barriers or facilitators to use (and, hence, enhance external validity and generalizability). One way of addressing this issue is through linked qualitative methods, including process evaluation, the value of which is increasingly recognized [19,60,61]. Another limitation is that we used the descriptions of the measures reported in the publications, and it was not always possible to ascertain what was included in the full instrument or details on the psychometric properties. Further studies to examine the quality of the instruments used would be helpful.

The preparation of this paper has been fruitful, in that it has confirmed evidence to support current IPDAS criteria for evaluating the effect of decision aids on the decision-making process and decision quality, and it has identified a number of questions that remain to be answered. Several questions emerged in our discussions, which we have summarized below as high-priority overarching questions for future research.

### How and when should the impact of PtDAs on the decision-making process and decision quality be measured?

Our review of the evidence found considerable variability in the constructs/attributes covered in trials of PtDAs and in the measurement instruments used. Our discussions noted the absence of an agreed-upon minimal set of “best” standardized, validated measures of the decision-making process and decision quality. There were gaps in the measurement of some attributes, which suggests a need for the development of new measurement instruments. Whether it is possible to have one instrument that could cover all the core decision making process attributes is not clear, but would be desirable. It will be important for this work to be done not only with reference to the core attributes, but also with careful consideration of the psychometric properties of measures devised to gauge those attributes. As researchers seek to develop new measures or strengthen existing ones, careful attention to strong clinical and psychometric properties is important [62].

Current measures are mostly patient self-reported measures, with a dearth of provider-reported, patient-provider interaction, or concordance measures. Levels of analysis (e.g., individual versus aggregate levels) need to be better specified in terms of the value of investment in decision quality measurement. Theoretical issues include differentiating between similar constructs and attributes when measuring and evaluating the impact of PtDAs, as well as identifying whether the focus of measurement should be around chosen or implemented options. For example, gaining a deeper understanding into the relative importance and role of patients’ subjective perceptions of the decision-making process (such as feeling informed or even feeling that the decision was shared with their provider), compared to more objective measures of knowledge or involvement, will be important to explore [63,64].

Additional questions concern specific effects of PtDAs. For example, in “informing” patients, how much and what type of patient knowledge is needed to support high quality decisions? Equally, what is the best time to measure the impact of PtDAs, in relation to concerns about hindsight bias (if measured too late) or other influences such as provider consultation (if measured too soon)?

### What are the other key constructs that should be measured to improve our understanding of the effectiveness of PtDAs?

This area of discussion focused on potential variants or extensions of existing PtDAs, and expansion of research on PtDAs to additional types of outcomes, settings, and populations. For example, in what situations is it more appropriate to use a briefer versus more complex PtDA? Are there certain patients or populations who should not get PtDAs? What are the active ingredients (mechanisms) of PtDAs and which are most essential or important to effectiveness? Given limited attention to potential harms/adverse effects of PtDAs, should harms such as bias, cognitive burden, or decision regret be measured, and, if so, how, when, and under what circumstances? Beyond evaluating the decision-making process and decision quality, what is the role of measurement of other factors such as treatment rates, service utilization, health inequalities/disparities, literacy, clinical outcomes, costs, and cost-effectiveness?

### How can we bring theory more directly into measurement?

Conceptual diversity exists in that multiple theories are relevant to the development of PtDAs and improving decision quality; e.g., decision-making theories, information-processing theories, and communication theories. The propositions of each theory may suggest different outcomes as priorities for evaluation. At present there is not an agreed-upon minimal set of evaluation measures in relation to how outcomes from each theory would be reconciled and/or linked to each other. There is also a need to assess if decision-making process variables are predictive of decision quality, and if so, how. Beyond measurement of the decision-making process and decision quality, increasingly PtDAs involve the consideration of options with a significant behavior change component—for example, surgery versus diet and exercise for obesity/weight management. In what way, if at all, does this behavior change component alter the approach to evaluating PtDAs? For example, is it also necessary to assess levels of self-efficacy and motivation in addition to knowledge and concordance?

A new initiative led by the US National Cancer Institute, using a web-based Grid-Enabled Measures (GEM) database, is collating constructs and measures relevant to shared decision making, including data on their development, psychometric properties, and availability [65]. The GEM-SDM database also allows peers to post informal reviews of the instruments, which will provide an important source of guidance to researchers in the field. This initiative has the potential to address some of the deficits identified in our review, such as providing a more comprehensive library of available instruments and, in the future, may help the field move toward consensus on a set of measures.

## Conclusions

In conclusion, this IPDAS-stimulated update of the evidence for the effectiveness of PtDAs has re-emphasized previous findings that PtDAS can improve the decision-making process and decision quality. Not only has evidence been strengthened, but also new evidence is emerging with respect to decision quality and the measurement of values-choice concordance.

Nonetheless, gaps remain, particularly with respect to measures of decision quality, which may be addressed by use of newly developed instruments in ongoing and future trials. Multiple measures continue to be used, particularly for measuring the quality of the decision-making process, with a lack of consensus on a set of core standard instruments. It will be important to work toward some level of harmonization of measures in order to enable better comparisons across studies. Finally, there are several important questions for future research and development in the area of measurement; we will need to tackle these issues in order to help the field advance.

## List of abbreviations used

API: Autonomy Preference Index; CPS: Control Preferences Scale; DCS: Decisional Conflict Scale; DSI: Decision Satisfaction Inventory; IPDAS: International Patient Decision Aid Standards; PDC: Perceived Decision Control; PICS: Perceived Involvement in Care Scale; PSDM: Problem Solving Decision Making Scale; PtDA: Patient decision aids; PDMS: Preparation for Decision Making Scale; SDMP: Satisfaction with Decision Making Process; SWD: Satisfaction with Decision

## Competing interests

The Informed Medical Decisions Foundation (IMDF) is a not-for-profit (501(c)3) private foundation (http://www.informedmedicaldecisions.org) that develops content for patient education programs. The Foundation has an arrangement with a for-profit company, Health Dialog, to co-produce these programs. The programs are used as part of the decision support and disease management services Health Dialog provides to consumers through health care organizations and employers.

KS receives salary and research support from IMDF.

DM and NW have also received research support from IMDF within the past five years.

CL receives salary support as Research Director for IMDF.

RT has been involved in developing PtDAs.

All other authors CB, JL, CN, MR, DS and CW have declared that they have no competing interests.

## Authors’ contributions

All authors contributed substantially to one or more of the following (1) the conception and design of study (all authors), acquisition of data or analysis and interpretation of data (all authors) (2) drafting the article or revising it critically for important intellectual content (KS, RT, DM, MR, CW) (3) final approval of the version to be submitted (all authors). The corresponding author, KS (ksepucha@partners.org) is responsible for the integrity of the work as a whole.

## Declarations

Publication of this supplement was partially funded by an unrestricted grant from the Informed Medical Decisions Foundation. The remaining costs were supplied by Karen Sepucha and Richard Thomson. Administrative and editorial support for this supplement was also provided by The University of Texas MD Anderson Cancer Center.

This article has been published as part of *BMC Medical Informatics and Decision Making* Volume 13 Supplement 2, 2013: The International Patient Decision Aid Standards (IPDAS) Collaboration’s Quality Dimensions: Theoretical Rationales, Current Evidence, and Emerging Issues. The full contents of the supplement are available online at http://www.biomedcentral.com/bmcmedinformdecismak/supplements/13/S2.
